# Discovering protein complexes in protein interaction networks via exploring the weak ties effect

**DOI:** 10.1186/1752-0509-6-S1-S6

**Published:** 2012-07-16

**Authors:** Xiaoke Ma, Lin Gao

**Affiliations:** 1 School of Computer Science and Technology, Xidian University, 710071, PR China

## Abstract

**Background:**

Studying protein complexes is very important in biological processes since it helps reveal the structure-functionality relationships in biological networks and much attention has been paid to accurately predict protein complexes from the increasing amount of protein-protein interaction (PPI) data. Most of the available algorithms are based on the assumption that dense subgraphs correspond to complexes, failing to take into account the inherence organization within protein complex and the roles of edges. Thus, there is a critical need to investigate the possibility of discovering protein complexes using the topological information hidden in edges.

**Results:**

To provide an investigation of the roles of edges in PPI networks, we show that the edges connecting less similar vertices in topology are more significant in maintaining the global connectivity, indicating the weak ties phenomenon in PPI networks. We further demonstrate that there is a negative relation between the weak tie strength and the topological similarity. By using the bridges, a reliable virtual network is constructed, in which each maximal clique corresponds to the core of a complex. By this notion, the detection of the protein complexes is transformed into a classic all-clique problem. A novel core-attachment based method is developed, which detects the cores and attachments, respectively. A comprehensive comparison among the existing algorithms and our algorithm has been made by comparing the predicted complexes against benchmark complexes.

**Conclusions:**

We proved that the weak tie effect exists in the PPI network and demonstrated that the density is insufficient to characterize the topological structure of protein complexes. Furthermore, the experimental results on the yeast PPI network show that the proposed method outperforms the state-of-the-art algorithms. The analysis of detected modules by the present algorithm suggests that most of these modules have well biological significance in context of complexes, suggesting that the roles of edges are critical in discovering protein complexes.

## Background

Interpretation of the completed biological genome sequences initiated a decade of landmark studies addressing the critical aspects of cell biology on a system-wide level, including gene expression analysis [1,2], gene disruptions detection [3,4], identification of protein subcellular location [5,6] and so on. An important and challenge task in proteomics is the detection of protein complexes from the available protein-protein interaction (PPI) networks generated by various experimental technologies such as yeast-two-hybrid [7], affinity purification [8], mass spectrometry [9], etc.

Protein complexes, consisting of molecular aggregations of proteins assembled by multiple protein interactions, are of the fundamental units of macro-molecular organizations and play crucial roles in integrating individual gene products to perform useful cellular functions. It is confirmed by the fact that the complex 'RNA polymerase II' transcribes genetic information into messages for ribosomes to produce proteins. Unfortunately, the mechanism for most of biological activities is still unknown and hence accurately predicting protein complexes from the available PPI data has a considerable merit of practice because it allows us to infer the principles of biological processes.

The general methods for protein complexes prediction are based on experimental and computational notions. Experimentally, the Tandem Affnity Purification (TAP) with mass spectrometry [9] turns out to be popular. However, it is far away from being a satisfying answer because of the limits on TAP [10]. For example, the transient low affinity protein complexes may be excluded because of the washing and purification operations in the TAP-MS. At the same time, this experimental approach needs the tag proteins to infer the protein complex. Gavin *et al*. [8] have indicated that only limited known yeast protein complex subunits can be extracted by the TAP-MS. Moreover, Schonbach [11] showed that, in order to validate the experimental results using the subcellular localization information, a preparation of subcellular fractionated lysates is a must. But the preparation procedure is time-consuming. That's why the computational approaches are becoming promising alternatives to complement the experimental ones.

Generally, protein interaction data can be effectively modeled as a graph (also called a network) by regarding each protein as a vertex and each known interaction between two proteins as an edge. Although there are plenty of related results in graph theory and many graph algorithms have been developed, it is still non-trivial to design an efficient algorithm to mine protein complexes from PPI networks. One reason is that there has not been an exact definition for a protein complex. To overcome this difficulty, Tong *et al*. [12] assumed that a protein complex corresponds to a dense subgraph since proteins in the same complex interact frequently among themselves, and similar discussion was also made in Ref. [13].

Although it is non-trivial to design effective and efficient computational methods for predicting complexes, many algorithms have been devoted to the issue. Markov Cluster Algorithm (MCL) [14,15] simulated random walks within graphs based on the intuition that a walker started at an arbitrary protein and visited a neighborhood vertex with a predefined probability. If he walked into a dense region, it is hard to get out of the region. Molecular Complex Detection (MCODE) [16] relied on the topological structure of a network, where it is assumed that a protein belongs to some complex if it has a subset of neighbors with high degree and there are many interactions among them. CFinder [17] defined a dense subgraph by using the concept of adjacent *k*-cliques. Other non-topological properties such as the functional information [18] and data of protein binding interface [19] are also incorporated into algorithms with an immediate purpose to improve the accuracy of prediction. In addition, there are some others relying solely on TAP data [20-22], which can be summarized as two points: first, a reliable PPI network is constructed by applying specific scoring strategies based on the purification records and selected protein interactions with high scores; second, some existing algorithms are employed to detect dense clusters in the newly constructed networks.

**Figure 1:**
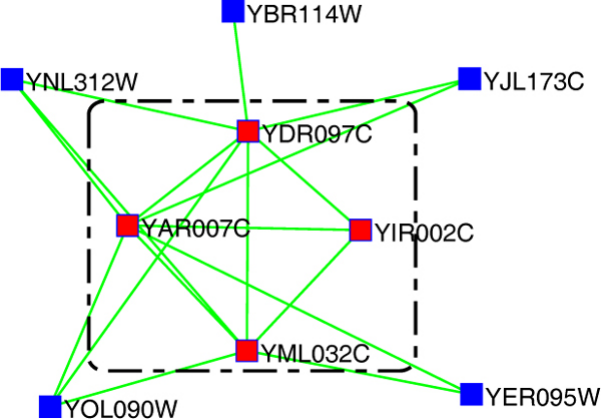
**An schematic example of core-attachment structure of protein complexes**. An example of the DNA repair complex [8], whose core consists of four red proteins in the dotted square and others are the attachments of this complex. The interactions in this figure are from the DIP data.

Except the biological information, some newly developed algorithms using the core-attachment structure in complexes revealed by Gavin *et al*. [8] (As shown in Figure 1). Leung *et al*. [23] proposed the CORE algorithm, a statistical framework to identify protein-complex cores. The probability for two proteins to be in the same protein-complex core is mainly determined by two factors: whether the two proteins interact or not and the number of their common neighbors. The CORE then calculates the p-values for all pairs of proteins to detect cores. Wu *et al*. [24] presented the Coach consisting of two steps: it first defines core vertices from the neighborhood graphs and then detects protein-complex cores as the hearts of protein complexes; it then includes the attachments into the cores to form biologically meaningful structures. Ma *et al*. [25] showed that the density of a subgraph is insufficient to characterize the complex and further demonstrated that the graph communicability is much better in characterizing the protein complexes. There are also many newly developed techniques for protein complex prediction [26-29]. Further information concerning the computational approaches for predicting protein complexes can be obtained from [30].

The core-attachment based approaches outperform dramatically the available state-of-the-art algorithms, demonstrating the significance of the structure and indicating the critical role of it in discovering protein complexes. This is one of the our major motivations. On the other hand, another major problem confounding the existing computational algorithm is that, available PPI networks are too sparse, for instance, the average numbers of interactions per protein are 5.29, 6.98, and 10.62 in DIP [31], Krogan [22], and Gavin [21], respectively. In these PPI networks, many protein complexes are difficult to be extracted since the sparse networks are full of noises [32]. Therefore, designing an efficient algorithm that gets rid of the noise is an important and challenging task to predict protein complexes. Unfortunately, previous algorithms did not pay enough attention to the problem since they only filter the noise by deleting nodes with degree 1 based on the fact that the interactions between proteins have lower reliability to the topological reliability measures [33,34]. Aside from issues of noise, all the existing computational approaches only make use of the topological structure information from the vertices and fail to take into consideration the roles of edges. It, however, is unreasonable to ignore the roles of edges, say the *weak tie theory *[35] and *percolation *[36], since an edge may play an important role in enhancing the locality or be significant in maintaining the global connectivity. For example, the famous weak ties theory indicates the job opportunities and new ideas are usually from persons with weak connections. Furthermore, the weak ties can be used to characterized the topological properties of networks such as the stability of biological functions [37], the accuracy of network structure prediction [38], the structure in mobile communication networks [39]. And the percolation characterizes the tendency to undergo a topological phase transition as the number of connections is progressively increased. Motivated by these observations, we pose the following question:

**Question: ***whether the roles of edges can be used in protein complexes detection*?

In this study, we aim to investigate the possibility to extract protein complexes by exploring the roles of edges and develop an affirmative answer to the above question. In detail, similar to the weak ties effects in mobile communication [39] and document networks [40], we prove complementary results on the PPI networks that is the edges connecting less similar nodes are more significant in maintaining the global connectivity. By using the weak ties and percolation, a reliable virtual network is constructed from the original PPI network, in which each maximal clique corresponds to a protein complex. A core-attachment based method is developed. To test the performance of the proposed algorithm, we applied it to the PPI networks. The experimental results on the yeast PPI network show that the proposed method outperforms DPClus [41], DECAFF [42], MCL [14], MCODE [16] and Coach [24]. Further, the analysis of detected modules by the present algorithm suggests that most of these modules have well biological significance in context of complexes, suggesting that the roles of edges are critical in discovering protein complexes.

## Materials and methods

The key idea behind our algorithm consists of three main steps: (1) verifying the existence of weak ties effect in PPI networks; (2) constructing a reliable network by exploring the roles of edges; and (3) identifying the protein complexes by using a core-attachment based method. We show them in turns.

### Weak ties phenomenon in PPI networks

A network consists of two basic elements: vertices and edges. Many measurements are developed to characterize the role of a node for structure and function including random walk-based indices [43], PageRank score [44]. In comparison, the study of the edge's role is less extensive.

Actually, edges in a network usually have two roles to play: some contribute to the global connectivity like the ones connecting two clusters while others enhance the locality like the ones inside a cluster. In social networks, the two roles are reflected as two important phenomena, being respectively the homophily [45] and weak ties effects [46]. Homophily demonstrates that connections are more likely to be formed among individuals with close background, common characteristics. On the other hand, the weak ties phenomenon shows that the less similar individuals are prone to be connected with weaker strength. These weak ties have important roles to play in maintaining the global connectivity. It has been proved that the weak ties phenomenon exists in the mobile communication [39] and document networks [40]. But, the weak ties effect for PPI networks remains to be tested.

To investigate the weak ties effects in PPI networks, we quantify how the topological structure changes according to an edge percolation process. In detail, if the weak ties effect exists in terms of topological similarity, the network disintegrates faster when we delete edges successively in an ascending order of the similarity than that in descending order. Similar to [40] two measures are employed to quantify how topo-logical structure changes when the edges are removed. The first one is the fraction of vertices contained in the giant component, represented by *R_GC_*. The second one is the normalized susceptibility, defined as

(1) $$\tilde{S}=\underset{s<s_{max}}{\sum }s^{\text{2}}/N,$$

where *s *is the size of a connected subgraph, *N *is the size of the whole network and the sum includes all connected components. An obvious gap occurs when the network disintegrates [47].

Prior to studying the weak ties, the bridgeness of an edge should be discussed. In [40] it is defined as

(2) $$B=\sqrt{C_{u}C_{\upsilon }}/C_{\left(u,\upsilon \right)},$$

where (*u*, υ) is the edge with *u, υ *being the endpoints, *C_u_*is the size of the maximal clique containing vertex *u *and *C*_(*u,*υ) _is the size of the maximal clique containing (*u*, υ). It, however, can not distinguish the bridges and non-bridges because it fails to take into account the difference between a pair of vertices. The bridggness value for each edge in a clique is 1 according to Eq.(2). It is unreasonable because intuitively the larger the size of a clique is, the lower the probability for some edge in the clique being a bridge is. For example, edges in 3-clique are more prone to be bridges than ones in 8-clique.

Actually, if (*u,*υ) is a bridge, the roles of vertex *u*,υ should differ greatly since they belong to various groups, indicating that they are dissimilar in topology. Therefore, a new bridgeness is defined as

(3) $$B_{\left(u,\upsilon \right)}=\left(1-J\left(u,\upsilon \right)\right)\frac{\sqrt{C_{u\backslash \upsilon }C_{\upsilon \backslash u}}}{C\left(u,\upsilon \right)},$$

where *J*(*u, υ*) is the Jaccard similarity, i.e., $J\left(u,\upsilon \right)=\frac{\left|N\left(u\right)\cap N\left(\upsilon \right)\right|}{\left|N\left(u\right)\cap N\left(\upsilon \right)\right|}$ with *N*(*u*) being the neighbors of vertex *u*, and *C_u\υ_*is the size of the maximal clique containing *u *without *υ*. The 1*- J*(*u*, *υ*) measures the dissimilarity between the pair of endpoints while the latter component quantifies the relation between the neigbors of two endpoints. The physical interpretation of Eq.(3) is that only these edges whose endpoints are less similar in topological and maintain the global connectivity are the bridges. Compared with Eq.(2), the new index is more reasonable, for example, for an edge in a *m*-clique is $\frac{2\left(m-1\right)}{m^{2}}$, which decreases as the size of a clique increases.

**Figure 2:**
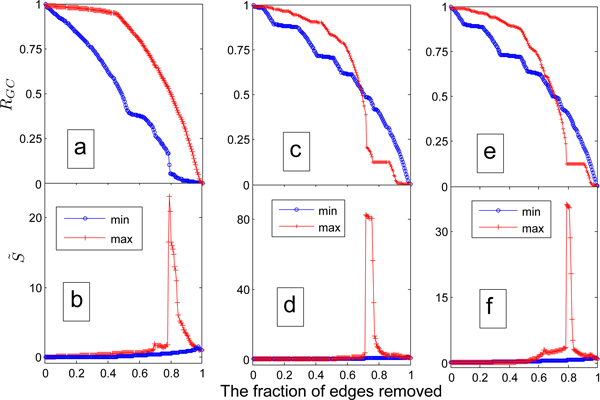
**Edge percolation results on PPI networks**. Plots (a) and (b) are for the topological similarity, while (c-d) and (e-f) are for bridgeness. In (a) and (b), the min- (max-) lines represent the processes where the edges are removed from the least (most) similar to the most (least) similar ones. In (c/e) and (d/f), the min- (max-) lines denote the processes where the edges with smaller (larger) bridgeness based on Eq.(3)/Eq.(2) are removed firstly.

Similar to Ref. [39], we quantify the weak ties phenomenon according to an edge percolation process. Generally speaking, if the weak ties phenomenon exists in terms of content similarity, the network will disintegrate much faster when we remove edges successively in ascending order of content similarity than in descending order. Figure 2 (a) shows *R_GC_* decreases much faster when the less similar edges are removed firstly. As shown in Figure 2 (b), a sharp peak occurs when the edges removed from the weakest to the strongest one, demonstrating the disintegration of the networks involved. Careful comparison of Figure 2 (a)(b) further shows that no percolation phase transition appears since there is no clear peak. These strongly supports the weak ties phenomenon in the PPI networks. In addition to the existence of weak ties phenomenon, we also have great interest in quantifying the edges' role of maintaining global connectivity. How good the bridgeness characterizes the weak ties phenomenon has been investigated in Figure 2 (c)(d). Figure 2 (c) indicates that *R_GC_* decreases much faster when the stronger bridges are removed firstly. As shown in Figure 2 (d), a sharp peak occurs when the edges removed from the strongest to the weakest one, demonstrating the disintegration of the networks involved. It is enough to assert that the bridgeness is an excellent alternative to describe the tie strength. To make a fair comparison between the index [40] in and ours, we also investigated how the networks changes in terms of bridgeness in Eq.(2) as shown in Figure 2 (e)(f). Compared Figure 2 (c)(d) with Figure 2 (e)(f), we can easily conclude that the network disintegrated more quickly (the bigger gaps in *R_GC_* and $\tilde{S}$) when the novel bridgeness is adopted, indicating that the new index is more efficient in characterizing the bridges in networks.

**Figure 3:**
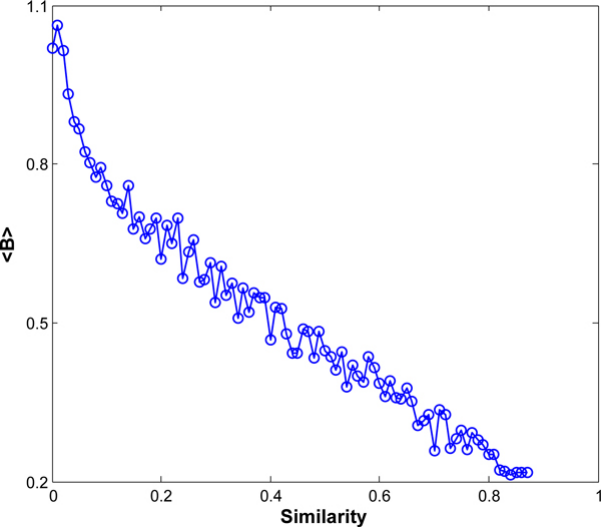
**Relation between bridgeness and topological similarity**. <*B*> is the average bridgeness values of edges with same topological similarity.

Furthermore, the relation between the topological similarity and bridgeness is also studied. The topological similarity for protein pair is defined as

(4) $$Sim=A+\beta A^{2}+\beta^{2}A^{3},$$

where *A *is the adjacency matrix of the network involved, (*A^k^*)_*ij *_denotes the number of walks of length *k *connecting vertex *υ_i_*and *υ*_*j*_, and *β *is parameter controlling the relevant importance of each component. The long walks receive greater weights when *β *> 1 while the short ones get more attention if *β *< 1. Here, we set *β *= 0.618. The result is showed in Figure 3. It demonstrates that there is a negative correlation between bridgeness and topological similarity, i.e., the weaker the similarity between a pair of proteins is, the stronger its bridgeness is.

### Constructing a reliable network

Gavin *et al *[8] have pointed out that the core of a complex has relatively more interactions while the attachments bind to the core proteins to form a biological complex, implying that the connectivity of a core is better than the whole complex.

To assess the topological proximity of a core, the measure of proximity of a pair of vertices should be handled beforehand. The most commonly used one is the graph distance, that is, the length of the shortest path connecting the pair of vertices. This quantity, however, is not appropriate for the biological networks largely because of two drawbacks: first, it does not take into account the local structural feature of the networks; second, it is very susceptible to the noises, e.g., a single missing edge effects the proximity, significantly. Thus, vertices connected by paths of various lengthes are likely to be functionality closer than vertices connected via a single path. In detail, give an edge, say (*u,υ*), it is reasonable to consider that the information transferred from *u *to *υ *through the right channels. The more the channels are, the better the connectivity is. Actually, in biological network, the genetic information is transferred by the pathways. From the aspect of graph theory, it is natural to consider the channels as various walks connecting *u*, *υ*. Likewise, we also take into consideration the strength of paths: the strength of the effect via longer paths with more intermediate vertices is very likely to be lower than those via shorter ones with fewer intermediaries. Given a walk of length *k*, say *υ*_1_*→υ*_2 _→ *... υ_k+_*_1_, its strength is defined as the product of the weights on each edge in the walk, i.e., $\prod_{i=1}^{k}w_{i,i+1}$ where *w_i, j_*is the weight on the edge (*υ_i_*, *υ_i_*_+1_).

Given an un-weighted PPI network, how to assign weights to edges is one of the key steps in our algorithm. As shown in Figure 3, there is a negative correlation between bridgeness and topological similarity. Thus, a novel strategy for the weight on the interaction (*u*, *υ*) based on the bridgeness in Eq.(3) is developed as

(5) $$D\left(u,\upsilon \right)=exp\left(-B_{\left(u,\upsilon \right)}\right).$$

The larger the bridgeness of an interaction is, the less weight it is.

Now, it is sufficient to deal with the similarity between a pair of proteins via various lengths of walks. (*D^k^*)_*u*υ _denotes the sum of strengths of all walks of length *k *connecting *u *and *υ*. Since the connectivity in cores is high, any pair of proteins in the same core should be tightly connected by short walks. Therefore, the similarity for a pair of proteins is the sum of strengths of walks connecting them, which can be a generalization of Eq.(4) as

(6) $$S=W+\beta W^{2}+\beta^{2}W^{3},$$

where *W *is a matrix with element (*W*)_*ij *_= *D*(*i, j*).

For any protein pairs, if the similarity between them is large enough, we have enough reason to believe they should be connected, otherwise, un-connected. Therefore, the proteins among a core should connect each other. To construct a virtual and reliable network for the original **PPI **network, similar to [25], a definition is proposed as

**Definition 1 ***The reliable network *Φ(*G, τ*) = (*V_τ_, E_τ_*) *for a PPI network G *= (*V, E*) *is the graph with V*_*τ*_=*V and E*_*τ *_= {(*u, υ*)│*u*, *υ *∊ *V, ψ*(*S_u,υ_,τ*) = 1}, *where ψ*(*x, τ*) *is a function defined as*

$$\psi \left(x,\tau \right)=\left\{\begin{array}{cc} 1 & \text{if}\;x\geq \tau ,\\ \text{0} & \text{otherwise}\text{.} \end{array}\right)$$

There are two good physic interpretations for Φ(*G, τ*): first of all, if the similarity of a pair of proteins is considered as the reliable score on the corresponding edge, Φ(*G*) can be considered as a reliable network of the original one; second, it can be understood as a perturbation of the original network by adding edges between vertices if there are enough short walks connecting them and deleting edges between vertex pairs if there are fewer short walks connecting them.

In this way, the core of a protein complex corresponds to a maximal clique in the virtual network. In the follows, we design algorithm to discover complexes by extracting cores and attachments, respectively.

### A core-attachment algorithm

The first task is to extract all the maximal cliques in the virtual network, known as the classic all cliques problem-an NP-hard problem [48]. Therefore, the exact algorithms are prohibited largely due to the complexity. The heuristic algorithms are selected in order to avoid the time issue. The Coach algorithm detects dense subgraphs very quickly and accurately from each vertex's neighborhood graphs [24]. We adopt the Protein-complex core mining algorithm in the Coach to identify approximately all cliques in the communicability graph Φ(*G*). Of course, others can be used to identify the cliques, for example, the greedy algorithm, the tabu search and so on.

What we would like to point out is that, although we adopt the same strategy to detect the cores, our algorithm differ greatly from Coach algorithm for two reasons: first, our algorithm detects core in a virtual network based on the weak ties phenomenon, while the Coach on the original network; second, the strategies for the attachment vary greatly.

Given a core denoted by an induced subgraph *G*(*U*) with *U *is the protein set of the core in the virtual network Φ(*G*), one crucial step to reveal the attachments is to construct the candidate protein set *CS*(*U*). For simplicity, we limit ourselves to only these proteins connected to at least one protein in *U*, i.e., *CS*(*U*) = {*v*│*υ *∊ *V *\ *U*, ∃*u *∊ *U *⇒ (*u,υ*) ∊ *E*}. What remains to be done is to determine the correct membership of each protein *v *in *CS*(*U*) by exploring the closeness between the vertex *υ *and *U*. If *υ *is an attachment of *G*_*U*_, there should be no protein *u*∊ *U *such that interaction (*u, υ*) is bridge. In other words, there must be many short walks connecting *υ *and vertices in *U*. Thus, we can define a new similarity function based on the brigdeness to quantify how closeness of a vertex *υ *to its core component as

(7) $$cl\left(\upsilon ,U\right)=\frac{\Sigma_{u\in U}S_{\upsilon u}}{\left|U\right|+1},$$

which quantifies the average closeness of *υ *to *U *from the aspect of connectivity. The larger *cl*(*υ, U*) is, the more walks connecting *υ *and the core. Thus, a vertex *υ *∊ *CS*(*U*) is selected as an attachment when the $cl\left(\upsilon ,U\right)\geq acl\left(U\cup N\left(U\right)\right)=\frac{\sum_{\upsilon \in N\left(U\right)}cl\left(\upsilon ,U\right)}{\left|N\left(U\right)\right|+\left|U\right|}$, indicating that the selected attachment has more connection ways with *U *than the average connectivity in *N*(*U*).

The procedure can be described as following:

Step 1: Compute the bridgeness for each interaction in PPI network *G *according to Eq.(3);

Step 2: Compute similarity matrix *S *based on Eqs.(5)(6);

Step 3: Construct the virtual network Φ(*G*) with a predefined threshold *τ*;

Step 4: Extract the cores using Protein-complex core mining algorithm [24];

Step 5: Detect the attachments for each core.

### Performance measures

The biological significance of the numerically computed modules can be validated by comparing the experimentally determined complexes (will be introduced in result section).

#### F-measure

Let *PS *(Predicted Set of Complexes) and *BS *(Benchmark Set of Complexes) be the sets of protein complexes that are predicted by a computational algorithm compared to the real complexes in the benchmark. *N_cb_*is the number of real complexes that match at least a predicted complex, i.e. *N*_*cb *_= │{*b*│*b *∊ *BS*, ∃*p *∊ *PS, NA*(*p, b*) *≥ t*}│, where *t *determines whether two sets match or not. *N_cp_*is the number of correct predictions that match at least one real complex, i.e., *N*_*cp *_= │{*p*│*p *∊ *PS,*∃*b *∊ *BS, NA*(*p, b*) *≥ t*}*│*. The F-measure can be used to quantize the closeness between two complex sets [20]:

(8) $$F=\frac{2\times Precision\times Recall}{Precision+Recall},$$

where $Precision=\frac{N_{cp}}{\left|PS\right|}$ and $Recall=\frac{N_{cb}}{\left|BS\right|}$[49].

#### Coverage rate

The coverage rate assesses how many proteins in the real complexes can be covered by the predicted complexes [50,51]. In detail, given the set of benchmark complexes *BS *and the set of predicted complexes *PS*, a │*BS*│ × │*PS*│ matrix *T *is constructed whereby each element *T_ij_*is the number of proteins in common between the *i-th *benchmarked complex and the *j*-th predicted complex. The coverage rate is defined as

(9) $$CR=\frac{\sum_{i=1}^{\left|BS\right|}\text{max}\left\{T_{ij}\right\}}{\sum_{i=1}^{\left|BS\right|}N_{i}},$$

where *N_i_*is the number of proteins in the *i*-th benchmarked complex.

#### P-value

The *P*-value [18] is employed. In detail, given a cluster *C *with *k *proteins in a functional group

*F*, the *P*-value is defined as

(10) $$P-value=1-\overset{k-1}{\underset{i=0}{\sum }}\frac{\left(\begin{array}{c} \left|F\right|\\ i \end{array}\right)\left(\begin{array}{c} \left|V\right|-\left|F\right|\\ \left|C\right|-i \end{array}\right)}{\left(\begin{array}{c} \left|V\right|\\ \left|C\right| \end{array}\right)},$$

where │*V*│ denotes the size of PPI network involved.

#### Geometric accuracy

To measure the robustness of the algorithm, the following measures are adopted [51]. Similar to Eq.(9), a matrix *T *is obtained by considering the annotated complexes as the *BS*. The clustering-wise sensitivity *Sn *is defined as

(11) $$Sn=\frac{\sum_{i=1}^{n}N_{i}{\text{max}}_{j}\left(T_{ij}/N_{i}\right)}{\sum_{i=1}^{n}N_{i}},$$

where *n*, *m *and *N_i_*are the sizes of *BS*, the number of clusters obtained by algorithms and the number of proteins in the *i*-th complexes, respectively. The positive predictive value *PPV *is defined as

(12) $$PPV=\frac{\sum_{j=1}^{m}\left(\sum_{i=1}^{n}T_{ij}\right)\overset{n}{\underset{i=1}{\text{max}}}T_{ij}/\sum_{i=1}^{n}T_{ij}}{\sum_{j=1}^{m}\sum_{i=1}^{n}T_{ij}}.$$

Based on *Sn *and *PPV*, the geometric accuracy is defined as

(13) $$ACC=\sqrt{SnPPV}.$$

#### Geometrical separation

Before our description about the geometrical separation, we define *separation*

(14) $$Sep_{ij}=F_{col_{ij}}PPV_{ij},$$

where $F_{col_{ij}}=\frac{T_{ij}}{\sum_{j=1}^{m}T_{ij}}$ Then, the geometrical separation *Sep *is defined as

(15) $$Sep=\sqrt{Sep_{co}Sep_{cl},}$$

where $Sep_{co}=\frac{\sum_{i=1}^{n}\sum_{j=1}^{m}Sep_{ij}}{n}$ and $Sep_{cl}=\frac{\sum_{i=1}^{n}\sum_{j=1}^{m}Sep_{ij}}{m}$.

## Results

In this section, the presented algorithm was applied to PPI networks with an immediate purpose to verify the performance from two perspectives: its ability to predict the protein complexes with accuracy, and the robustness of the algorithm. The algorithm was coded using MATLAB version 7.11.

### Data

The Database of Interaction Proteins [31] (DIP)(http://dip.doe-mbi.ucla.edu/[version yeast20071104]) data is adopted, which consists of 4,928 proteins and 17,201 interactions. To evaluate the protein complexes predicted by our algorithm, a benchmark set was constructed from the the MIPS [52], Aloy et al. [53] and the SGD database [54] based on the Gene Ontology (GO) notations, which consists of 428 protein complexes [50].

### F-measure and coverage rate

To further verify the novel bridgeness, we proposed two versions of our algorithm: Type I using the bridgeness in Eq.(2), Type II in Eq.(3). The basic information of predictions by various compared algorithms is summarized in Table 1. From it, the MCL identifies 1116 complexes, of which 193 mach 242 real protein complexes; DPClus extracts 1143 complexes, of which 193 match 274 real complexes, DECAFF detects 2190 protein complexes, of which 605 match 243 ones and Coach reveals 746 complexes, of which 289 match 249 real ones. Our Type I algorithm predicts 686 protein complexes, out of which 242 match 198 real ones in the benchmark, while Type II discovers 604 protein complexes, out of which 230 match 220 real ones in the benchmark.

**Table 1:** The results of various algorithms using DIP data

	MCL	DPClus	DECAFF	Coach	Our method-I	Our method-II
Predicted complexes	1116	1143	2190	746	686	620
Covered proteins	4930	2987	1832	1832	1776	1702
*N_cp_*	193	193	605	285	242	230
*N_cb_*	242	274	243	249	198	220

**Figure 4:**
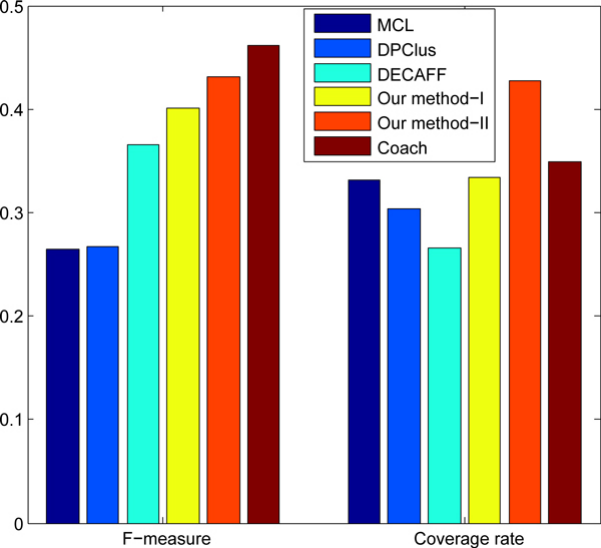
**F-measure and Coverage rate**. The performance comparison for various algorithms on DIP data.

Figure 4 shows the overall comparison in terms of F-measure and coverage rate on the DIP data. Although it is 2.9% lower than Coach algorithm, the F-measure of our algorithm Type II is 43.2%, which is 16.7%, 16.5% and 6.0% higher than MCL, DPClus and DECAFF, respectively. It demonstrates that our algorithm can predict protein complexes very accurately. From Figure 4, it is very easy to see that our method obtains the highest coverage rate of 42.8%, which is 7.9%, 9.6%, 11.4% and 16.2% higher than Coach,MCL, DPClus and DECAFF, respectively. It shows that the predicted complexes by our algorithm can cover the most proteins involved in the real complexes. From Figure 4, we can make a conclusion that our algorithm is obviously outperform the MCL, DPClus and DECAFF, and it makes a better balance between the F-measure and Coverage rate than the Coach. Compared Type I with Type II, we discovered that the Type II is much better than Type I, demonstrating that the efficiency of the proposed bridgeness. Such results further demonstrate that the critical phenomenon in the PPI can be used for enhancing the prediction accuracy.

### P-value

To further investigate the biological significance of the predicted complexes, the *P*-value is adopted here. The functional homogeneity *P*-value is the probability that a given set of proteins is enriched by a given functional group merely by chance, following the hypergeometric distribution. It is the probability of cooccurrence of proteins with common functions. Accordingly, a low *P*-value of a predicted complex indicates that the collective occurrence of these proteins in the complex does not merely combine by chance and thus achieves high statistical significance. The values are calculated by the GO::TermFinder [55].

We discarded all clusters with *P*-value above a cutoff threshold. In the experiments, we chose a cutoff of 1 *× *10^-2 ^for each protein complex because it offers a compromise between complex-cluster matching rate and a clustering passing rate.

Table 2 shows the comparison results in terms of the proportion of significant protein complexes over all predicted ones. In the Table, our method-II achieves the best performance (83.7%), implying the majority of predicted complexes are significant. Furthermore, the Coach has a comparative performance with our algorithm but the MCL and DPClus can only predict a small proportion of significant complexes. To further demonstrate the predicted protein complexes, 5 protein complexes with very low *P-*values, predicted by our method. The second columun is Table 3 refers to the ratio of the annotated proteins to ones in the identified complex.

**Table 2:** Statistical significance of protein complexes obtained by various algorithms on DIP data

	MCL	DPClus	DECAFF	Coach	Our method-I	Our method-II
Predicted complexes	1116	1143	2190	746	686	620
Significant complexes	312	352	1653	622	536	519
Proportion (%)	34.2	30.8	75.5	83.4	78.1	83.7

**Table 3:** Selected complexes predicted by our method-II on DIP data

ID	Match	*P*-value	Predicted complexes				Function
1	90.5%	5.44E-44	YBL002W	YBR009C	YBR154C	YDL140C	DNA-directed RNA polymerase activity
			YDL150W	YGL070C	YJR063W	YKL144C	
			YKR025W	YNL113W	YNR003C	YOR116C	
			YOR151C	YOR207C	YOR210W	YOR224C	
			YOR341W	YPR010C	YPR110C	YPR187W	
			YPR190C				
2	94.4%	8.77E-40	YDL150W	YKL144C	YKR025W	YNL151C	RNA polymerase activity
			YNR003C	YOR116C	YOR207C	YPR110C	
			YBL002W	YBR154C	YDR045C	YJR063W	
			YNL113W	YOR224C	YOR341W	YPR010C	
			YPR187W YPR190C				
3	100%	7.57E-26	YPL138C	YDR469W	YBR175W	YHR119W	histone methyltransferase activity (H3-K4 specific)
			YBR258C YAR003W YKL018W YLR015W				
4	88.2%	1.49E-20	YBL093C	YBR253W	YDR443C	YNL025C	transcription regulator activity
			YNL236W	YOR140W	YBR193C	YCR081W	
			YDL005C	YER022W	YGL151W	YGR104C	
			YHR041C YOL051W YOL135C YPL042C YPL248C				
5	100%	2.64E-21	Q0085 YBL099W YDR298C YDR377W YJR121W				proton-transporting ATPase activity, rotational mechanism
			YKL016C YML081C-A YPL078C YPR020W				

### Size and density distributions

Because the above experiments are sufficient to prove that the superiority of the proposed bridgeness, we only focused on the Type II method in the forthcoming experiment.

**Figure 5:**
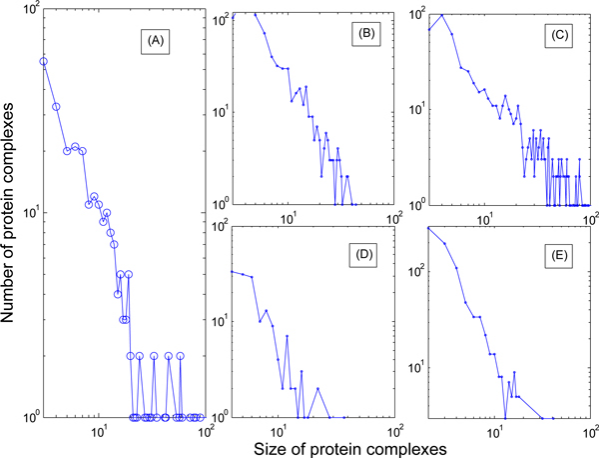
**Size distribution of predicted complexes**. Protein complex size distribution of various method and the benchmark set (A) the benchmark set; (B) the Coach; (C) our algorithm; (D) the DPClus; (E) the MCL.

The *P-*values of predicted complexes by our algorithm support that the roles of interactions in PPI networks is promising on enhancing the accuracy of prediction. The module size distribution of predicted protein complexes for each compared methods on the DIP network has been shown in Figure 5. From it we can conclude that the major trend generated by our algorithm is very similar to that of the complexes in the benchmark set, which suggest that the definition of protein complex based on the weak tie effect is reasonable. However, the Coach can identify much less modules than these in the benchmark set, and its trend is different from that of the benchmark set. What we would like to point out is that the size distributions of the DPClus and MCL algorithms are very different from the previous ones.

**Figure 6:**
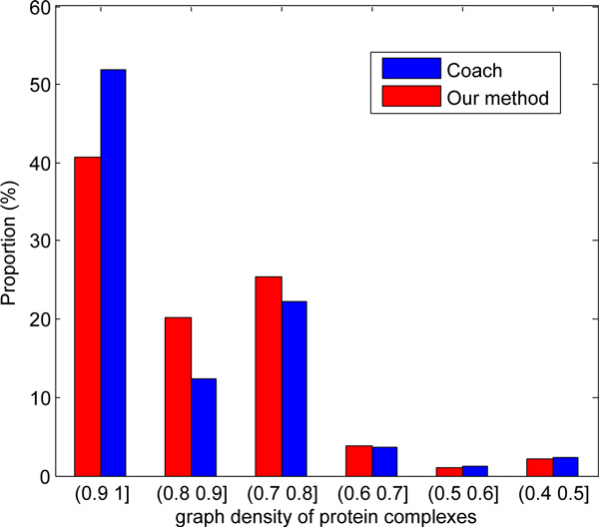
**Density distribution of predicted complexes**. The comparison on the density of predicted protein complexes from various algorithm.

Notice that our algorithm is quite different from those based on discovering the dense subgraphs because it makes use of the weak ties effect. To verify the difference on the densities of the predicted complexes, we compared the Coach algorithm with our method in terms of the graph densities of the predicted complexes, shown in the Figure 6. It is easy to figure out that more than 50% complexes predicted by the Coach algorithm whose densities are more than 0.9, while only 40% complexes predicted by our method whose densities are larger than 0.9. Furthermore, our algorithm can discover more protein complexes whose densities in range [0.6 0.9], which suggests that the density is not the only manner to characterize the protein complex and others are necessary and reasonable.

### Effects of the parameters

**Figure 7:**
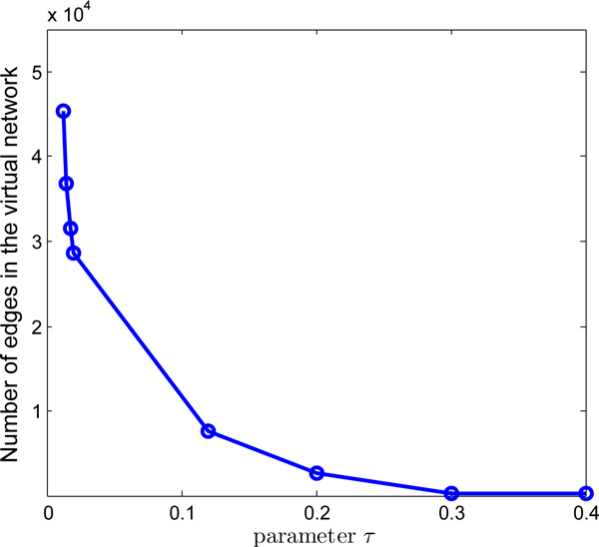
**Effect of parameter *τ***. The plot of the number of edges in the virtual network for various values of *τ*

This subsection is devoted to investigate how the parameters *τ *and *β *used effects the performance. The value of *τ *controls the size of a core, the total number of cores in the virtual graph, and the connectivity 'strength' of the network involved. Therefore, we studied its effect on the size of the virtual network. Figure 7 shows how the number of edges in the virtual network changes for various values of *τ*. From it, we can see that the size of the virtual graph decreases dramatically when the value of *τ *increases from 0 to 0.4. Specifically, the size is approximately 3 × 10^4 ^if *τ *= 0*.*02. The reason is that when the value of *τ *increases, only the edges whose connectivity is strong enough are maintained.

**Figure 8:**
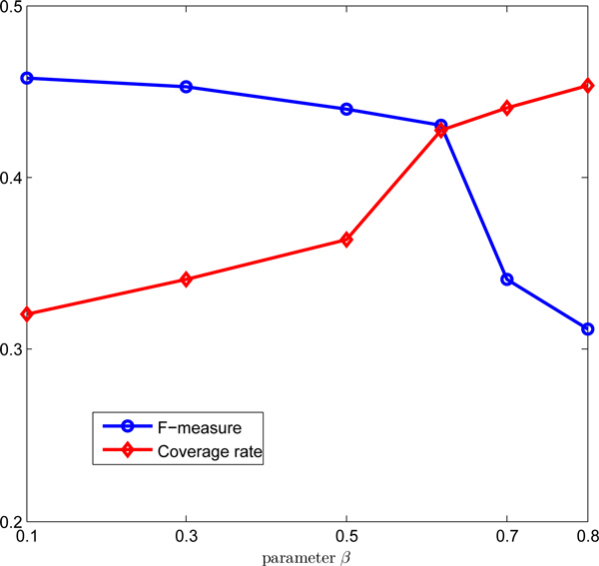
**Effect of parameter *β***. The plot of the F-measure and Coverage rate for different values of *β*.

The parameter *β *controls the weights on the edges. Thus, we study its effect on the accuracy of prediction in terms of F-measure and coverage rate. Figure 8 demonstrates that the F-measure decreases, while the coverage rate increases when *β *increases. A possible reason is that the size of a maximal clique in the virtual network decreases when *β *increases, resulting in many small cores by dividing the large cores in the virtual graphs with small *β*. As *β *increases, more and more proteins in the PPI data are covered because the number of predicted protein complexes increases. For this reason, the coverage rate keeps increasing. To make a good balance between the F-measure and coverage rate, we set *β *= 0.618.

### Robustness analysis

The robustness analysis on the proposed algorithm was discussed in this subsection. The benchmark networks adopted here originated from Ref. [51]. In detail, from the protein complexes annotated in the MIPS database [52], an interaction network named a *test graph *is constructed by regarding each protein as a vertex and connecting each pair of nodes in the same complexes. The test graph has a poor value for assessing the robustness of the algorithms because each protein complex corresponds to a clique in the test graph. To solve this problem, the *altered graphs *are constructed from the test graph by adding or deleting the edges in various proportions. For the sake of convenience, the altered graph is denoted by *AG_add, del_*where *add *and *del *show the percentage of added and deleted edges, respectively.

In this experiment, only the MCL and Coach algorithms are selected for a comparison. The reason is that it is reported that the MCL is the most robust algorithms [51], and the Coach algorithm is the best core-attachment based method.

**Figure 9:**
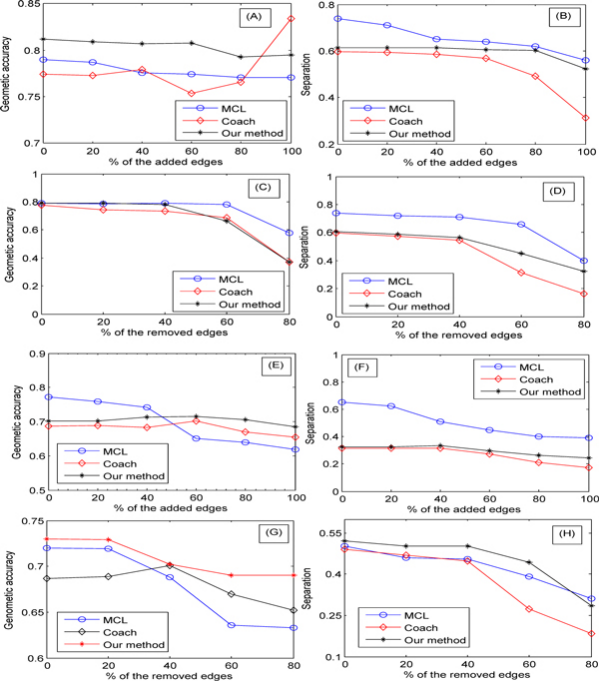
**Robustness analysis**. In the left panel, each curve denotes the value of accuracy, while that in the right represents the value of separation: (A-B) edge addition to the test graph; (C-D) edge removal from the test graph; (E-F) edge addition to the altered graph with 40% of edges removed randomly; (G-H) edge removal from the altered graph with 40% of edges added randomly.

The Figure 9(A) shows how the geometric accuracy fluctuates as the number of edges increases. Increasing proportions of edges were randomly added to the test graph from 0% to 100%. Both the MCL and our algorithm are barely affected by the additions of up to 100% edges, while the performance of Coach is acceptable for low values of noise, they change dramatically when the percentage of added edges increases to 40%. A good reason is that as the percentage of added edges increases, the added edges connecting to the vertices in different cliques yield larger complexes(through merging the small complexes). In this case, the altered graph is not suitable for correctly extracting the complexes by the Coach algorithm. However, our algorithm can remove the noise dramatically because it extracts the protein complexes in a virtual network, where some of the added edges are filtered by increasing the value of the threshold *τ*.

Figure 9(B) displays the impact of edge addition on the separation. We can see that both the MCL and our algorithm have good performances when the percentage of the added edges increases to 80%, while the performance of the Coach algorithm decreases when the percentage of added edges increases to 20%. The impacts of edge removals on the geometric accuracy and separation are shown in Figure 9(C)(D), respectively. Figure 9(C) demonstrates that both the MCL and our algorithm outperform the Coach algorithm. A possible reason is that, as more and more edges are deleted, it becomes more and more difficult to re-obtain the deleted edges. When the percentage of removed edges is more than 20%, the virtual network constructed by our algorithm differs greatly from the original test graph. The general trends in Figure 9(D) are similar to those displayed in Figure 9(C).

Figure 9 (A-D) are the results on the networks being either added or removed edges, while Figure 9 (E-H) are the results on the networks involving both addition and removal. Figure 9 (E) demonstrates the effect of edge addition on the altered network from which 40% of the edges have been deleted previously. From it one can easily draw a conclusion that, when the addition less than 50%, the MCL outperforms the Coach and our algorithm, but when the the addition greater than 50%, both methods outperform the MCL. There is a good explanation: since the Coach and our algorithm are clique-based method, edge deletion destroys the structure of cliques, decreasing their performance; when more and more edges are added, some of the cliques destroyed previously are recovered, enhancing their performance. Furthermore, these two algorithms are barely affected by addition that is up to 100%, as the MCL decreases significantly the edges start to increase gradually. The values of separation on this type of altered network are shown in Figure 9 (F), where the MCL is at its the best performance. However, both the Coach and our algorithm are more stable than the MCL. The results on edge deletion on the altered network from which 40% of the edges have been added previously are shown in Figure 9 (G-H), which are similar to those in Figure 9 (E-F).

## Conclusions

Protein complexes are key and basic molecular units in cellular functions and computational approaches to discovering accurately the unknown protein complexes hidden in the available PPI data are critical need. At present all these computational algorithms focus on the roles of proteins without taking into account the roles of interactions.

In this paper, we investigate the possibility to predict protein complexes with the roles of edges in PPI networks. Firstly, the weak ties phenomenon in the PPI network is proved by using the concept of bridge. Secondly, a reliable and virtual PPI network is constructed making use the relations of topological similarity and bridgeness. Finally, a core-attachment algorithm is designed. The experimental results demonstrate that the roles of edges in biological network is more promising than the roles of proteins, implying the significant importance of the roles of interactions.

The possible future research directions are

*• *Because biological network is a special kind of social networks, to uncover the social behaviors hidden in biological networks and make the most of them to discover biological problems, such as protein complex prediction, disease causing genes prediction, are very promising.

*• *The discovery of structure-functionality is a hot and very important topic in bioinformatics. How to associate the social behaviors including the weak ties with the functions is challenge and critical since it provides a deep insight into the biological processes.

Thus, designing effective and efficient methods which can solve these problems will be very important and interesting.

## Competing interests

The authors declare that they have no competing interests.

## Authors' contributions

XM designed the study. XM and LG implemented the method, performed the experiments, analyzed the data and wrote the manuscript.
